# Time in Bed, Sleeping Difficulties, and Nutrition in Pregnant New Zealand Women

**DOI:** 10.3390/nu15051130

**Published:** 2023-02-23

**Authors:** Barry William McDonald, Patricia Ellyett Watson

**Affiliations:** 1 School of Mathematical and Computational Sciences, Massey University, Auckland 0632, New Zealand; 2 Institute of Food, Nutrition and Human Health, Massey University, Auckland 0632, New Zealand

**Keywords:** time in bed, sleep, sleeping difficulties, physical activity, pregnancy, trimester, diet, water, nutrient intake, New Zealand

## Abstract

We consider the relationship between time in bed (TIB) and sleeping difficulties with demographic variables and nutrient intakes in the second (T2) and third (T3) trimester of pregnancy. Data were acquired from a volunteer sample of New Zealand pregnant women. In T2 and T3, questionnaires were administered, diets were obtained from one 24 h recall and three weighed food records, and physical activity was measured with the use of three 24 h diaries. In total, 370 women had complete information in T2 and 310 in T3. In both trimesters, TIB was associated with welfare or disability status, marital status and age. In T2, TIB was associated with work, childcare, education and pre-pregnancy alcohol consumption. There were fewer significant lifestyle covariates in T3. In both trimesters, TIB declined with increasing dietary intake, especially water, protein, biotin, potassium, magnesium, calcium, phosphorus and manganese. Adjusted for weight of dietary intake and welfare/disability, TIB declined with increasing nutrient density of B vitamins, saturated fats, potassium, fructose and lactose; and TIB increased with carbohydrate, sucrose and vitamin E. Subjective sleeping difficulties increased with the week of gestation, morning sickness severity, anxiety, dairy and saturated fat intake, and they decreased with fruit, vegetable and monounsaturated fat intake. The study highlights the changing influence of covariates throughout the pregnancy and corroborates several published findings on the relationship of diet and sleep.

## 1. Introduction

There has been much recent interest in the relationship between diet and sleep, with several broad-based reviews [1,2,3,4,5,6,7]. The evidence shows that those with short sleep duration tend to consume more energy from fats [8,9], while high-carbohydrate (CHO) diets tend to be associated with longer sleep. Evidence regarding protein is mixed, and a recent review has concluded it has little effect on sleep [10]. The relationship between diet and sleep duration is thought to be bidirectional [1,4,6] and possibly non-linear, with optimal sleep duration (neither too short nor too long) being associated with healthier diets [11,12]. The timing of nutrient intake (chrononutrition) is also important [13,14].

The focus of sleep studies varies. Some observational studies (usually large samples) concerning sleep and diet focus on sleep duration—for example, Refs. [12,15,16]. Other studies consider a number of different aspects of sleep quality, of which duration is only a part: characteristics such as overall time in bed (TIB), sleep latency (time between going to bed and falling asleep), sleep efficiency (proportion of bed time actually asleep), frequency of awakening, or sense of not feeling refreshed after a night’s sleep [17,18,19].

The methods of measuring sleep also vary. Laboratory-based studies using polysomnography are considered the gold standard for comprehensive measurement of sleep quality [2], but they are usually limited to small samples, such as those in intervention studies—for example, Ref. [20]. Wrist-mounted actigraphs are now a popular alternative for moderate-sized samples, again allowing accurate and objective measurement of a number of sleep characteristics [16,17,21]. An activity diary is another objective night-by-night measure of TIB or sleep, although inevitably with more error than electronic measurement. Activity diaries have the virtue of serving large samples with minimal cost. For example, Ref. [22] considers activity in 2454 children and adolescents, reported by a parent or caregiver, based on two 24 h activity diaries for one randomly chosen weekday and one randomly chosen weekend day (Ref. [17] recommends that the averages be based on more night-by-night measures).

Other studies measure sleep based on a subjective estimate of average sleep over a week or month. Larger studies tend to measure sleep duration through a question, such as “How much sleep do you usually get at night on weekdays or workdays” (NHANES question) [23], or similar [12,14,15]. The responses may then be categorized in hours. Sleep quality is frequently assessed using the Pittsburgh Sleep Quality Index (PSQI) [24], which considers seven aspects, with a total score that is usually dichotomized to distinguish good and poor quality sleep. Poor quality sleep tends to be associated with very short or very long sleep duration and chronic health problems [4,24,25].

There has been limited research on the relationship between diet and sleep specifically in pregnant women. In the GUSTO study of pregnant Singaporean women [26], data from a 24 h recall were used in a healthy eating index and an analysis of dietary pattern as defined by principal components analysis. The researchers found that a healthier diet was associated with better quality sleep, as measured by PSQI, but did not find a relationship between diet and sleep duration. A study of pregnant African American women [27] found shorter TIB being associated with higher intake of fruit and vegetables, and diet variables were related to sleep timing (midpoint of time asleep). A study of pregnant overweight or obese women [17] found that those with a more pro-inflammatory diet (high in sugar, fats, ultra-processed and fast foods) differed significantly in two aspects of sleep quality from those with anti-inflammatory diet (high in fruits, vegetables, fish and grains); namely, they had significantly longer sleep latency and some evidence of a longer period of lying in bed after waking. A study of pregnant Australian women [28] found those with diets with a high percentage of energy from CHO and monounsaturated fatty acids (MUFA) had poorer sleep quality, but there was no difference found for duration. Although not focusing on diet, a study [29] found women who were overweight or obese prior to pregnancy and who then had excess gestational weight gain (indicative of higher energy intake) tended to have shorter sleep duration and more sleep disruption.

The study of diet and sleep in pregnancy is complicated by changes that occur over the course of pregnancy. It is well recognized that sleep quality (though not necessarily duration) tends to deteriorate in the third trimester [30,31]. A study of Saudi women [32] found that PSQI rose (i.e., quality deteriorated) in the second and third trimester compared to the first trimester, and sleep duration was longer in late pregnancy. That study found that women with low income, low serum vitamin D, high energy intake and long periods of sitting down were particularly likely to find their sleep quality deteriorating over the course of the pregnancy.

On the other hand, there is mixed evidence of a change in diet over pregnancy. The GESTAFIT study of Spanish women [33] found that third-trimester women had higher intakes of fruits, vegetables and whole dairy products but no other significant changes in intake or in adherence to a Mediterranean food pattern; their overall conclusion was that food behavior did not change over pregnancy. A study of overweight and obese American women [34] found no significant difference in fat intake or fruit and vegetable intake across the three trimesters of pregnancy. By contrast, the GUSTO study of Singaporean women [35] found increasing consumption of milk, fruit and vegetables and decreasing intake of tea, coffee, soft drinks and seafood as the pregnancy progressed.

There have been other studies in young-to-midlife women, where pregnancy was not a factor. A study of 80 young Japanese women [21] examined dietary intake and quality and their association with sleep efficiency, as measured by actigraphy. They found that energy intake, protein intake and intakes of vitamin K and B2 and several minerals were lower in a group with low sleep efficiency. A much larger sample of midlife Mexican women [36] identified dietary patterns based on a food frequency questionnaire, with sleep quality measured by PSQI. They found that those following a healthier diet tended to have better sleep quality. A study of 92 non-pregnant female Saudi students found high polyphenol intake to be a protective factor against poor sleep [37]. A study of Iranian women and infants post-partum [38] found that better quality diet—as measured by the dietary approaches to stop hypertension (DASH) criterion—was associated with better quality sleep according to the PSQI.

The present study considered the relationship between nutrient intake and TIB in New Zealand (NZ) pregnant women in the second and third trimesters and the effect of associated covariates. TIB was used as the response because the data were gleaned from 24 h activity diaries, and not all women distinguished sleep from bed rest. We also examined whether there was any association with subjective difficulty sleeping. The purpose was to find evidence that confirms the literature—or not—on the relationship of nutrient intake with sleep or TIB and to suggest new associations to be explored in future research.

## 2. Materials and Methods

This article considers data from the “Nutrition in Pregnancy” study collated by the authors at Massey University, NZ [39]. The ethical approval for the study was obtained from the Massey University Human Ethics Committee and the Auckland Ethics Committee North Health. The funding was provided for 500 subjects, with selection biased toward a greater proportion of NZ Māori or Pacific Island Polynesian women and women of lower socioeconomic status than in the general population. Subjects lived in rural and urban centers in the upper North Island of NZ and were volunteers recruited mostly through advertising in free child-health clinics or religious or community facilities throughout the study area, with some recruited by media advertising and word-of-mouth. As the aim was to provide a representative snapshot of nutrition in the pregnant population, there was no preliminary dietary information given to the volunteers, but they were supplied with personalized information after the data were collected. In total, 504 women around the 14th week of pregnancy were recruited.

Subjects were matched to an interviewer of their own ethnicity. Interviewers had some qualification in nutrition or community health and were trained to administer questionnaires in an unbiased way to elicit 24 h dietary recall data, obtain physical measurements (e.g., skinfolds) and to train the subjects to fill out 24 h dietary records and activity diaries. The interviewers visited each subject at a place of her choosing near the start of month 4 (second trimester—T2) and month 7 (third trimester—T3) of her pregnancy and also post-partum. Questionnaires were administered in the subject’s preferred language to determine demographic, medical, health and lifestyle details. The questionnaires were similar in content and format to those used to determine this information in NZ national nutrition surveys [40], for which statistical reliability and validity had been established. The study questions and protocols were assessed by independent nutritional experts, ethics committees and approved by the NZ Ministry of Health who funded the study.

Maternal height, weight and triceps, biceps and costal skinfolds were measured using calibrated standard equipment according to the procedures set out by Gibson [41]. Gestational age was calculated from the last date of menstruation. The severity of morning sickness was ascertained by a questionnaire, coded as: None (0), Nausea occasionally (1), Nausea few hours/day (2), Nausea and vomit few hours/day (3), Nausea all day (4), Nausea all day, vomit occasionally (5), Vomit all day (6), Hospitalized (7).

Dietary intake was assessed in both T2 and T3. The interviewer administered a 24-hour dietary recall, followed by a 3-day food record kept by the subject. In the recall, the interviewer used numerous aids to assess the weight or volume of each food and beverage portion consumed in the previous 24 h. After the recall interview and training, subjects recorded in their preferred language all food and beverage portions consumed over 3 days using the measuring cups and spoons provided to assess the volume ingested. Days were not necessarily consecutive; every four days of diet assessment included one weekend day. Foodworks, utilizing the NZ Food Composition database (NZ Institute for Plant and Food Research), was employed to calculate the nutrient intake for each woman each day. Subsequent analysis of variance of nutrient intake found no significant difference overall between the 24-hour recalls and 3-day food records in T2 and T3 diets (*p =* 0.099, Pillai’s test), nor were there significant differences in nutrient density between the methods (MANOVA *p =* 0.158). Therefore, the 24-hour recall data were combined with the 3-day diet record data to generate a mean intake of each nutrient for each woman in T2 and T3. In addition to beverages, tap water intake was also estimated in the questionnaires in T2 and T3 by recall without aids to estimate the volume, and hence, the values are less accurate than for dietary water [42].

Around the same time in T2 and T3, subjects completed three 24 h physical activity diaries. The diaries had squares for every 10 min, from 9 a.m. one day to 9 a.m. the following day, although subjects could start at any time of day. The diaries did not have to be consecutive days. Subjects recorded in each square what they were doing for most of the time in that 10 min period. Subjects were prompted that “if you were doing anything for a long period of time, e.g., sleeping, just write ‘sleep’ in the first square and an arrow to when you finished sleeping”. However, some wrote “bed”, for example, so our data were unable to distinguish actual sleep from lying down at rest. As there may be a difference in activity between T2 and T3, this article considers the relationship between minutes of overall TIB and nutrient intake in each trimester separately. The T2 and T3 figures represent two different snapshots of (in most cases) the same individuals but using separate nutrient and TIB data each month (76% of T2 respondents and 91% of T3 respondents feature in both analyses).

Data were checked using standard statistical techniques (e.g., crosstabs, scatterplots). Minitab 19 was used for statistical analysis. TIB was found to vary widely according to work and health status, etc. Therefore, to reduce the effect of outliers on the analysis, the most extreme 1.5% of short durations and 2.5% of long down durations were winsorized (replaced by 420 and 780 min, respectively). The latter cutpoint (13 h) is abnormally long for sleep alone and may indicate health problems, but the range of times still gives quantitative insight into individuals who need more or less sleep or bed rest. The winsorized TIB durations were used as the dependent variable in bivariate and multiple linear regressions. Nutrient intakes were analyzed on a log-to-base-2 scale, so that regression slopes can be interpreted as the effect of doubling the intake. Nutrient density (intake per MJ of energy consumed) was also considered.

The variables considered as possible covariates included maternal anthropometric measures, ethnicity, socioeconomic measures, work hours, family, lifestyle and childcare details, morning sickness, alcohol consumption prior to pregnancy and gestational age at the time of interviews We could not adjust for all possible confounders because of our relatively small sample; instead, we first identified significant non-nutritional covariates in simple regression and then tried them in a multiple regression model for TIB. The maximal multiple regression model with all significant variables (*p* ≤ 0.05) was chosen, but to ensure no effect was missed, all reasonable alternative variables were re-examined for any significant predictors. The finalized regression models were based on cases with complete data for the included predictors. The nutrients discussed below are those that were significant in the presence of significant background covariates and remained significant when other nutrients were included.

In addition, the T2 interview included some questions on typical weekly and daily consumption of broad food groups. One question was “How many times a week do you eat…?” with the following food groups: Breads/Cereals; Fruit; Vegetables; Milk/Dairy; Meat/Alternatives; Takeaways. The response options were: Daily (coded 4); 3–4 times a week (3); 1–2 times a week (2); Rarely (1); Never (0). A follow-up question asked “How many times a day do you eat…” with the same food groups as above and with the subject’s open-ended responses converted into the number of servings. The coded values were considered as covariate values in a regression model.

The questionnaire also had a question relevant to subjective sleep quality. In both T2 and T3, subjects were asked “Do you have any of the following:”, which listed a variety of symptoms or medical conditions, including “Difficulty sleeping”. Subjects could choose from the following options: Never (coded 0), Rarely (coded 1), Sometimes (2) and Often (3). The answers to this question were analyzed as the dependent variable in an ordinal logistic regression model. Other symptoms, including nausea, constipation, frequent urination and others, were considered as potential covariates in a regression model. The questionnaire further asked “How have you been feeling lately?” with the following headings: Full of energy; Tired; Calm and Peaceful; Worn Out; Happy; Anxious; Fit and Well; Depressed. The response options in each case were: All the time (coded 4); Most of the time (3); Some of the time (2); A little of the time (1); and None of the time (0). The coded responses were considered as covariate values in a regression model.

## 3. Results

Actual gestation at the T2 questionnaire averaged 21.2 weeks (standard deviation SD 5.2) and at the T3 questionnaire, 30.5 weeks (SD 2.9). Dietary information was collected for 96% of women at T2 and 88% at T3. The T2 physical activity diaries were completed by 73% of subjects, and T3 diaries by 62%. Compliance at T2 was higher (82%) among those in the top seventy percent of household incomes than those in the lower income group (56%) and higher among Europeans and Asians (85%) than among Polynesians (46%).

Median TIB was 585 min (9.75 h) for women in T2 (*n* = 371) and 597 min (9.96 h) at T3 (*n* = 310). This difference was not large (12 min) but significant (*p* = 0.043, paired *t*-test). The minimum was 305 min (5.1 h) and maximum 1115 min (18.6 h). To reduce the effect of such outliers on regressions, the bottom 1.5% of times and top 2.5% were winsorized to 420 and 780 min. This still enables an investigation of significant trends that separate long and short sleepers. There was no association between TIB and the proportion of out-of-bed time spent in high-energy-expenditure activities, such as sport/exercise or vigorous housework, or between TIB and the proportion of time in sedentary activities.

### 3.1. Demographic and Non-Dietary Variables

As shown in Table 1, there was a significant decrease in TIB in T2 with increasing age (2.2 min less per year, *p* = 0.007), years of high school education (13.4 min less per year, *p* = 0.004) and household income level (*p* = 0.028). In T2, women in paid work averaged 29 min less TIB than those not in paid work (*p* = 0.001), or 0.7 min less per 1 h of paid work (*p* = 0.002), but by T3, there was no difference (*p* = 0.771). Those dependent on government welfare payments averaged 48 min more TIB than the others (*p* < 0.001). Rural women averaged 24 min more TIB than urban women in T2 (*p* = 0.015), but the difference did not persist through to T3. Three women were disabled, which was too few for significance, even though they averaged 108 min more TIB than non-disabled women. Married women had significantly less TIB than unmarried women (37 min in T3, *p* = 0.001), and there was a similar difference for presence of any live-in partner (40 min at T3), but the latter difference was not significant (*p* = 0.087), possibly due to small numbers of un-partnered women. There was no significant difference in TIB between nulliparous women and those with a child/children, but there was a marginal decrease in TIB with the number of preschoolers (children under 5 years); this became significant in multiple regression. TIB decreased marginally with increasing actual number of gestational weeks at T2 measurements (this became significant in multiple regression) but not at T3. There was no relationship between TIB and current morning sickness, but there was a marginal increase in TIB with the frequency of nausea (*p* = 0.075) and constipation (*p* = 0.071) (a subsequent table shows that difficulty sleeping was significantly related to the severity of morning sickness). On the other hand, women who felt full of energy in T3 spent significantly less TIB (*p* = 0.003). No association was found between TIB and being a smoker, BMI or frequency of feeling anxious or depressed, but there was an increase in TIB with the thickness of biceps skinfold (*p* = 0.045). Finally, in T2, there was an association with usual (pre-pregnancy) alcohol consumption; those who usually drank beer averaged 28.5 min longer TIB than those who did not (*p* = 0.004), while those who usually drank wine averaged 21.8 min less TIB than those who did not (*p* = 0.010). A similar association was found with the quantity of beer and wine consumed (square root of usual g of beer or wine). No bivariate association was found for the usual consumption of spirits (hard liquor), but in a multivariate model, the usual consumption of spirits pre-pregnancy was associated with less TIB at T2. Few women admitted drinking alcohol during pregnancy, and so, consumption during pregnancy was not a significant covariate of TIB.

These results suggest that any study of sleep or TIB in pregnancy needs to take account of the gestational age of the fetus at the time of study, as both the sleep duration (or TIB) and the relevant covariates may vary throughout the course of pregnancy. Specifically, covariates related to pre-pregnant lifestyle may still affect sleep or TIB in early pregnancy but have diminished importance in later pregnancy.

### 3.2. Relationship of Individual Nutrient Intakes with TIB

Table 2 shows that significant relationships exist between nutrient intakes and the winsorized TIB in both T2 and T3. Nutrients are analyzed on the log-to-the-base2 scale, which means that the slopes are interpretable as the change in minutes of TIB associated with a doubling of nutrient intake; for example, a woman who consumed twice the total water (in food, beverages and plain water) as another woman in T2 is estimated to spend 31 min less TIB than the other woman. The *p*-values tend to be smaller in T2 due to more data. The most significant predictors of decreasing TIB in both trimesters were increasing water consumption (*p* < 0.001) and increasing dietary intake (weight of food and beverages) (*p* < 0.001). The dry weight of intake and overall energy intake were not significant, although several individual nutrients (protein, thiamine, riboflavin, biotin, potassium, magnesium, phosphorus, calcium, manganese and copper) were significantly (*p* < 0.05) negatively related to TIB in both trimesters. In addition, T2 showed a significant decline in TIB with increasing glucose, fructose, pantothenate, niacin, folate and zinc, while T3 showed a significant decline with increasing lactose.

The analysis shows that there are a large number of nutrient intakes potentially related to TIB. Some of this will be due to nutrient intakes being correlated. In the next subsection, we therefore consider nutrient density after adjusting for log2 (dietary weight).

### 3.3. Significant Relationships between TIB and Individual Nutrient Densities

Table 3 only shows the significant relationships between nutrient densities and TIB. Nutrient density is defined here as log2 (nutrient intake/total energy in MJ), and it is a measure of diet quality. To adjust for diet quantity, log2 (dietary intake) is included in the regression. High dietary intake is related to shorter TIB. Table 3 also adjusts for whether or not the woman was dependent on government welfare payments (such as unemployment or sickness benefit) or was disabled; as mentioned in Section 3.1, women in this category averaged significantly higher TIB.

Table 3 shows the relationships for significant individual variables. TIB was significantly longer in T2 if the diet was rich in carbohydrate, and sucrose in particular, and significantly shorter if the diet was rich in thiamin, riboflavin or niacin. In T3, the duration of TIB was shorter for diets rich in saturated fats. For example, doubling the sucrose density corresponded to an additional 16.6 min TIB in T2, while doubling the SFA density corresponded to 28 min less TIB in T3.

### 3.4. Multivariate Models for TIB

Table 4 shows multiple regression models for TIB in each trimester.

In T2, around month 4, there are several confounders, which help explain the variation in TIB: longer times with welfare/disability status (*p* = 0.002); shorter times with hours of paid work (*p* = 0.005), actual week of gestation (*p* = 0.022), number of preschool children (*p* = 0.001) and whether the woman usually drank spirits prior to pregnancy (*p* = 0.011). The last confounder may be related to societal differences, as well as to the alcohol itself. Longer TIB was associated with higher nutrient density of total sugars (*p* = 0.012) and vitamin E (*p* = 0.008) but negatively related to dietary intake (*p* < 0.001) and nutrient density of fructose (*p* = 0.008) as well as potassium (*p* = 0.002).

By T3, most confounder variables no longer significantly explain the variation in TIB, with the exception of welfare/disability status (*p* < 0.001). Shorter TIB was associated with higher dietary intake (*p* < 0.001) and diets rich in saturated fatty acids (*p* < 0.039). The proportion of variation explained by confounders and diet is much lower in late pregnancy.

### 3.5. Dietary Pattern Analysis for Nutrient Density

A factor analysis was carried out on the nutrient density variables to identify the possible associations between dietary pattern and TIB.

Using the same confounders as in Table 4, and adjusted for log2 (dietary intake), only one marginally significant dietary pattern (factor) was discovered (*p* = 0.071) based on nutrient densities. In T2, this factor loaded positively on CHO and total sugars, especially sucrose, glucose and fructose, and vitamin C, and negatively on total fats, SFA, MUFA, PUFA, starch and sodium and chlorine. Women at two standard deviations above the mean for this factor (high sugar diet) were in bed an estimated 29.5 min longer than women at two standard deviations below the mean (high fat, salt and starch diet).

Similarly, in T3, only one marginally significant factor was discovered (*p* = 0.070). This factor was almost the same as in T2, except that the negative end also loaded heavily on cholesterol, retinol, vitamin A and B12 but not on starch. Women at two standard deviations above the mean for this factor (high sugar diets) were in bed for an estimated 34 min longer than women at two standard deviations below the mean (diets high in fats, salt and other animal products).

### 3.6. Association with Subjective Sleeping Difficulties

In both trimesters, subjects were asked “Do you have any of the following”, with Difficulty Sleeping as one of the symptoms. Subjects could respond as follows: Never (0), Rarely (1), Sometimes (2), Often (3). Table 5 summarizes the responses. Sleeping difficulties increased as the pregnancy proceeded; the median category changed from Rarely (1) to Sometimes (2). A paired *t*-test showed the mean change in response code was 0.58 (SE 0.05, 95%CI 0.48 to 0.68, *p* < 0.001). This indicates decreasing sleep quality over the course of pregnancy.

We consider an ordinal logistic regression for Difficulty Sleeping. Note that negative coefficients correspond to increasing difficulty sleeping. Table 6 shows that, in T2, difficulty sleeping increased significantly with the week of gestation (*p* = 0.005), severity of morning sickness (*p* = 0.018), frequency of anxiety (*p* = 0.033) and depression (*p* < 0.001), intake of niacin (*p* = 0.008) and a high intake of SFA (0.008) and low intake of MUFA (*p* = 0.002) (these last could be expressed as a ratio of SFA/MUFA, *p* = 0.004). If food group frequencies are used in place of dietary intake, T2 sleeping difficulties increased with typical daily servings of dairy (*p* = 0.042) but decreased with daily servings of vegetables (*p* = 0.029).

Table 7 shows that, in T3, the severity of morning sickness earlier in the pregnancy continued to predict sleeping difficulties (*p* < 0.001), as did (independently) whether symptoms of morning sickness were still continuing at T3 (*p* = 0.044). Sleeping difficulties continued to be related to anxiety (*p* = 0.002), but depression was not significant. Sleeping difficulties increased with higher ratio of vitamin B6 to dietary weight of intake (*p* = 0.001) but decreased with intake of β-carotene (*p* = 0.016). In terms of food group frequencies (these were only collected in T2), sleeping difficulties in T3 still increased with typical weekly dairy intake (*p* = 0.020) and decreased with weekly fruit intake (*p* = 0.008).

## 4. Discussion

### 4.1. TIB vs. Time Sleeping

This paper differs from most in using TIB as the response variable instead of time asleep. This is because it was a secondary analysis of activity diary data. A different sample [43] of 197 NZ pregnant women showed those with less TIB averaged larger babies and less maternal weight gain post-partum, while those with greater daily activity levels tended to have higher wellbeing, longer gestation and less probability of the infant needing admission to a neonatal intensive care unit.

TIB values, however, are larger than time sleeping, which can make the interpretation difficult. For self-reported sleep, the commonly used categories are <5 h for very short, 5–<7 h for short, 7–<9 h for normal and ≥9 h for long sleep (Ref. [25]). An actigraphy study [29] of sleep duration in overweight or obese US women in late pregnancy gave a mean (SD) of 419 (88) min (around 7 h) per night and 88 (55) min of sleep during the day. A study of urban African American pregnant women [27] reported a mean (SD) TIB of 8.6 (2.1) h. By contrast, our median TIB was 585 min (9.75 h) for women in T2 and 597 min in T3 (as a comparison, Ref. [43], based on a sample with a larger proportion of rural women, reports medians of 605 and 622 for “sleep/lie down”, i.e., TIB, in T2 and T3).

Part of the difference between TIB and sleep duration may be due to reading or watching television in bed, or non-sleeping bed rest due to morning sickness or ill health. Indeed, some daytime TIB was probably included—c.f. the mean 88 min daytime sleep reported for pregnant women in Ref. [29]. Thus, the intrinsic difference between TIB and sleep duration makes direct comparison of times difficult. On the other hand, the trends may be similar; variables that are significantly correlated with sleep duration may also be significantly correlated with TIB and vice versa. We look for this similarity of trends.

### 4.2. Association of TIB with Demographic Variables

In our study, the bivariate analysis found that TIB was negatively associated with age, household income, education and presence of a partner, and positively associated with frequency of anxiety and depression. However, none of these associations remained significant in multiple regressions. Shorter TIB in the second trimester was found, in multiple regression, to be significantly associated with work and family responsibilities (especially with preschool children), while rural women, those on very low income (welfare) or those with disabilities tended to have longer TIB. Our data did not show any association between TIB and BMI or ethnicity. There was a trend of shorter TIB with actual week of gestation in T2, but the weekly trend was no longer significant by T3.

There is limited literature relating demographic variables to sleep duration or TIB in pregnant women [32]. The small sample of urban African American pregnant women [27] reported non-significant trends of less TIB among older women, those married and those with a college degree. Another US study of pregnant women [44] found lower sleep duration if they were older and had graduated high school (but not college graduates), but, contrary to our findings, less so if they were unmarried, unemployed and on low income (the contradiction may be partly explained by different levels of welfare support in NZ compared to the USA). Ref. [45] reports unemployment, low income and low education to be predictive of poor sleep quality (in terms of PSQI). A study of Saudi women [32] found that high income was somewhat preventive against the deterioration in sleep quality and duration over the course of pregnancy.

### 4.3. Association of TIB with Nutrients

We found no association of TIB with total energy intake but a negative association with total dietary intake, and in particular, total water and dietary water (moisture). Others [25,46] have found a similar association with water intake. The type of beverage is important; a study of NHANES data [47] with self-reported sleep durations found those in the short sleep group (≤5 h) consumed more sugar-sweetened beverages than a reference group (7–8 h), while longer sleepers (≥9 h) consumed significantly less coffee and less plain water than the reference group. A study [48] of sweetened beverages in children similarly found shorter sleep duration in those consuming soft drinks at least daily. Along related lines, we found TIB decreased significantly with increasing glucose and fructose in T2 and with lactose in T3.

TIB decreased significantly with increasing protein (considered on its own) but not total fat or total CHO. However, after adjusting for total weight of dietary intake, the effect of protein became no longer significant. After adjusting for dietary intake, TIB was found to increase significantly with diets relatively dense in CHO and sucrose in T2 (variables considered individually). In a multivariate model, this relationship was clarified as increasing TIB with increasing density of total sugars but decreasing density of fructose. Thus, it appears that diets rich in fructose tend to result in shorter TIB, while other types of CHO tend toward longer TIB.

In T3, it was found that, after adjusting for dietary intake, TIB decreased significantly with the increasing density of SFA. Ref. [9] found short sleep duration was associated with fat intake generally. It was not possible to produce a regression model that was simultaneously significant for densities of sugars and densities of fats, since these effects are negatively correlated. Dietary pattern analysis came close to resolving the issue, indicating that, based on principal component (PC) analysis, women with relatively high sugar diets (on one end of the PC scale) tended toward longer TIB, while women with diets relatively high in fats and animal product diets (on the other end of the PC scale) tended to have shorter TIB. However, the significance of the PC was only marginal. A study of adolescents [16] similarly found shorter sleep duration being associated with more calories from fats and less from CHO. A study of Turkish adults [19] also identified high SFA as being associated with short sleep duration.

There were a large number of micronutrients individually negatively correlated with TIB, notably biotin and other B vitamins, potassium, magnesium, calcium, phosphorus, manganese. After adjusting for dietary intake, the nutrient densities of niacin, riboflavin and thiamin were individually negatively associated with TIB in T2. The multivariate model in Table 4, also adjusted for density of sugars and fructose, found TIB was positively related to nutrient density of vitamin E and negatively related to density of potassium. The last result accords with a randomized controlled trial [49], which found shorter sleep duration among men receiving potassium supplements. Therefore, there is something there, but the correlations among nutrients make this a confusing picture, and it will take a larger sample to sort the effects out. In particular, some researchers (e.g., Refs. [11,19]) have found that many nutrients had a U-shaped relationship with sleep duration.

Our results are restricted to overall linear associations, not U-shaped relationships. In those terms, our study indicates that shorter TIB is broadly associated with better nutrition. In the words of an anonymous reviewer: “*Although causality cannot be inferred, it seems that women who are getting adequate nutrients may need less resting time, and thus are spending less time lying in bed (while potentially still getting adequate amounts of sleep)*”.

### 4.4. Sleeping Difficulties

No association was found between TIB and the severity of morning sickness, but those women with more severe morning sickness had a higher frequency of sleeping difficulties in both T2 and (interestingly) T3, when most women no longer had symptoms.

Our data showed the frequency of women experiencing sleeping difficulties was lower with increased intake of vegetables (T2) and fruit and beta-carotene (T3), but sleeping difficulties were more frequent with increased intake of dairy products in both trimesters. Given that fruit and vegetable intakes are correlated, this concurs reasonably well with the GESTAFIT study [33], which found sleep quality (measured by PSQI) was better with increasing fruit intake in the second trimester and increasing olive oil intake and adherence to a Mediterranean food pattern in the second and third trimester. Similarly, our study showed the frequency of sleeping difficulties increased with intake of SFA in T2 and vitamin B6 in T3, which compares with the GESTAFIT finding of worse sleep quality with increasing red meat and subproducts (significant) and poultry (not significant) intakes. The small study of African American pregnant women [27] reported that shorter TIB was associated with higher intake of fruits and vegetables but (somewhat contradictorily) also of pastries.

The study of Turkish adults [19] identified high CHO, beta-carotene, vitamin E, thiamin, vitamin B6, vitamin C, calcium, magnesium, potassium as being associated with good sleep quality. Our finding of less sleeping difficulty with increasing beta-carotene confirmed that of Ref. [19], but our multivariate model did not allow us to confirm the other findings. An experimental study [50] found B6 supplementation to have little effect on sleep, but subjects given a high-dose B complex supplement had lower self-rated sleep quality and were significantly more tired on waking. Several researchers (e.g., Ref. [32]) have considered the relationship of vitamin D and sleep quality, but our data did not show any relationship. Ref. [34] found sleep disturbances being associated with dietary fat intake but short (better) sleep latency being associated with higher fruit and vegetable intake. Our data also showed MUFA intake being associated with fewer sleeping difficulties, which confirms a finding [28] that higher MUFA intake is associated with improved sleep quality in pregnancy.

### 4.5. Alcohol

Alcohol consumption has been shown to be associated with poorer sleep quality in observational [51,52] and clinical settings [53]. A large community study [51] found a dose–response relationship between alcohol consumption and worse sleep quality six years later and that after adjusting for confounders, “consumption of hard liquor [spirits] but not beer or wine, was significantly associated with poor sleep quality”. By contrast, Ref. [52] found increasing alcohol consumption was associated with shorter sleep duration in young males but not in a small sample of females. Our study found a positive association between the amount of pre-pregnancy beer and TIB, a negative association for wine and, in multiple regression, that a history of pre-pregnancy consumption of spirits was associated with shorter TIB. However, we did not find an association between alcohol consumption and subjective frequency of sleeping difficulties. These findings may be confounded by sociodemographic factors associated with differences in the choice of beverage. The findings suggest that the association of alcohol with TIB, sleep duration and sleep quality may be different for beer, wine and spirits, and therefore, the alcohol type must be considered in a nuanced way.

### 4.6. Limitations

A limitation of this study is that the focus of data collection was on the activity level rather than sleep alone; therefore, the durations of sleep and bed rest were conflated into TIB, which may also include daytime rest. The use of a wrist-mounted actigraph would give more accurate data. Another limitation is that our study did not separate weekend and weekday sleep, which can give rise to different results, e.g., “social jetlag” [54]. Future studies should routinely collect this information. Additionally, TIB was obtained from only three days of activity diary each trimester, and unlike in Ref. [22], the days were not pre-assigned. Collection over more nights spanning a week would be an advantage [17]. It may not be too much of a burden on research participants if they were asked to diarize the week’s day-to-day sleep measurements. If complete 24 h activity diaries were required, this would have a high compliance burden or require actigraphs. Actigraphs themselves require cost and time for dispersal and retrieval. The difficulty of comparing TIB with average sleep estimates is discussed in Section 4.1.

This study did not separately consider the factors related to long TIB. Long TIB probably reflects poor sleep quality, i.e., longer sleep latency and longer time in bed after waking. Some researchers (e.g., Ref. [25]) look for variables related to both short and long sleep categories compared to a baseline (central) category. We did not analyze the factors associated with longer TIB because we felt such times would be excessively confounded by health issues (such as lying in because of morning sickness) or behavioral matters (such as reading or watching television in bed), for which we had no data. Not analyzing long TIB separately does make the interpretation of relationships more difficult, as the comments in this paper are, perforce, restricted to significant overall linear trends and not U-shaped relationships between TIB and nutrient intakes.

Another limitation is that sleeping difficulties are measured by a single four-category variable, which may be less reliable than asking several questions. The validated PSQI questionnaire [24] would have given a more faceted approach to sleep quality than our simple question of the frequency of “difficulty sleeping”. We would recommend PSQI for future research. Both our question and PSQI rely on subjective choice of response by the subject.

The high degree of correlation among the nutrients makes the interpretation of the nutritional effects difficult. Some controlling for correlation was performed by adjusting for weight of dietary intake and using nutrient density as a covariate. However, in a survey of free-living adults, it is not possible to eliminate all associations between the nutrients. This is where experimental studies are very valuable, as diets can be adjusted to increase or decrease specific nutrients and measure whether this specific adjustment has a biological consequence.

Despite these limitations, our study was sufficiently sensitive to: confirm several trends of the relationship between diet and sleep applicable to TIB; explore the differences in TIB and the confounders based on the trimester of pregnancy; and suggest some associations, which can be investigated in future research on sleep.

### 4.7. Conclusions

In summary, this study highlighted the changing influence of covariates throughout the pregnancy and found the demographic and nutritional covariates of TIB and sleeping difficulties, which corroborate several published findings on the relationship between diet and sleep.

## Figures and Tables

**Table 1:** Demographic and non-dietary predictors of minutes of TIB per day in the second and third trimester.

Covariate	Second Trimester Coefficient (SE)	*p*-Value	Third Trimester Coefficient (SE)	*p*-Value
Age (years)	−2.23 (0.83)	0.007 **	−2.54 (0.92)	0.006 **
Gestation (weeks)	−1.56 (0.91)	0.088	1.27 (1.63)	0.434
Current morning sickness (T2 yes = 40%, T3 yes = 10%)	3.8 (8.4)	0.654	13.4 (15.2)	0.377
Smoker (yes = 10%)	15.0 (14.0)	0.284	11.5 (15.9)	0.467
Number of preschoolers	−9.9 (5.8)	0.088	−9.5 (6.1)	0.118
Married (yes = 78%)	−23.2 (10.0)	0.021 *	−37.9 (11.3)	0.001 **
Any partner ( yes = 95%)	−11.5 (19.5)	0.555	−40.1 (23.4)	0.087
In paid work (yes = 65%)	−29.1 (8.6)	0.001 **	−3.0 (9.8)	0.763
Weekly hours of paid work	−0.70 (0.22)	0.002 **	−0.26 (0.26)	0.312
Dependent on welfare payments (yes = 10%) ^a^	47.8 (13.3)	<0.001 ***	52.8 (14.3)	<0.001 ***
Household income level ^b^ (low = 1, high = 4)	−11.2 (5.1)	0.028 **	−18.5 (5.2)	<0.001 ***
Years of high school (≤2, 3, 4, ≥5)	−13.4 (4.6)	0.004 **	−10.5 (5.4)	0.052
Rural (yes = 22%)	24.2 (9.9)	0.015 *	−3.8 (11.5)	0.743
Biceps skinfold (mm)	1.09 (0.54)	0.045 *	0.28 (0.62)	0.658
BMI at measure	−0.09 (0.93)	0.927	0.34 (1.04)	0.741
Nausea ^c^	6.8 (3.8)	0.075	8.6 (5.5)	0.118
Constipation ^c^	7.2 (4.0)	0.071	3.3 (4.8)	0.496
Frequent Urination ^c^	0.7 (3.8)	0.861	−2.8 (4.9)	0.571
Full of energy ^c^	−1.5 (4.3)	0.729	−12.8 (4.4)	0.003 **
Anxious ^c^	5.7 (4.0)	0.213	3.3 (3.5)	0.341
Depressed ^c^	8.6 (5.7)	0.131	−4.9 (4.2)	0.246
Usually Drank Beer ^d^	28.5 (7.8)	0.004 **	4.0 (11.2)	0.720
Usually Drank Wine ^d^	−21.8 (8.5)	0.010 *	−13.1 (9.6)	0.171
Usually Drank Spirits ^d^	−10.0 (10.1)	0.342	7.9 (11.8)	0.501
Sqrt Usual Beer (g) ^d^	0.79 (0.23)	0.001 **	0.47 (0.27)	0.083
Sqrt Usual Wine (g) ^d^	−1.15 (0.56)	0.038 *	−1.00 (0.65)	0.124
Sqrt Usual Spirits (g) ^d^	0.03 (0.97)	0.975	1.06 (1.16)	0.361

* *p* < 0.05, ** *p* < 0.01, *** *p* < 0.001. ^a^ In receipt of unemployment, sickness or domestic purposes benefit and a community services card for subsidized health care. ^b^ Household income level 1 = lowest 4%, 2 = next 7%, 3 = next 11%, 4 = remaining 76% of households. ^c^ Coded on scale 0 = None of the time, 1 = A little of the time, 2 = Some of the time, 3 = Most of the time, 4 = All the time. ^d^ Before pregnancy. No relationship was found between TIB and alcohol consumption during pregnancy.

**Table 2:** Relationship between log2 (nutrient intakes) and minutes of TIB in the second and third trimester; each nutrient is considered on its own.

Nutrient (All on log2 Scale)	Second Trimester (*n* = 370)			Third Trimester (*n* = 307)		
	Slope (SE)	R^2^ (%)	*p*-Value	Slope (SE)	R^2^ (%)	*p*-Value
Dietary Weight ^a^ g	−42.7 (10.3)	4.44	<0.001 ***	−41.2 (11.3)	4.17	<0.001 ***
Dry Weight g	−20.5 (18.0)	0.72	0.103	−22.1 (12.9)	0.96	0.087
Dietary Water ^a^ g	−39.3 (9.2)	4.77	<0.001 ***	−38.0 (10.1)	4.43	<0.001 ***
Total Water ^a^ g	−31.4 (7.1)	5.01	<0.001 ***	−36.0 (11.9)	2.97	0.003 **
Total Weight ^a^ g	−35.1 (8.0)	4.95	<0.001 ***	−28.0 (9.3)	2.98	0.003 **
Energy kJ	−16.4 (12.2)	0.49	0.180	−25.0 (8.3)	0.87	0.102
Protein g	−22.2 (9.9)	1.36	0.025 *	−25.1 (10.7)	1.79	0.019 *
Total Fat g	−9.4 (9.0)	0.30	0.293	−14.2 (9.1)	0.79	0.121
Carbohydrate g	−12.9 (11.9)	0.32	0.277	−9.7 (11.9)	0.06	0.413
Total Sugar g	−6.0 (8.6)	0.13	0.484	−9.3 (8.0)	0.45	0.242
Glucose g	−16.9 (6.4)	1.84	0.009 **	−6.1 (5.7)	0.36	0.292
Fructose g	−15.9 (6.1)	1.83	0.009 **	−5.3 (5.6)	0.29	0.343
Sucrose g	5.3 (6.6)	0.17	0.425	−4.7 (6.7)	0.16	0.481
Lactose g	−7.9 (4.4)	0.86	0.074	−11.6 (3.9)	2.75	0.004 **
Maltose g	3.0 (5.6)	0.08	0.593	−2.8 (5.7)	0.08	0.624
Starch g	−13.6 (10.7)	0.44	0.203	−3.8 (11.7)	0.03	0.746
Dietary Fiber g	−14.5 (8.1)	0.86	0.074	−13.1 (9.0)	0.69	0.147
Cholesterol mg	−4.9 (6.5)	0.15	0.456	−8.0 (7.0)	0.43	0.254
SFA ^b^ g	−5.9 (8.2)	0.14	0.470	−14.9 (8.2)	1.05	0.072
MUFA ^b^ g	−11.1 (8.2)	0.49	0.177	−13.0 (8.5)	0.76	0.128
PUFA ^b^ g	−4.4 (6.8)	0.12	0.513	−4.0 (7.4)	0.10	0.584
B-carotene µg	−3.6 (4.0)	0.22	0.371	−2.2 (4.5)	0.08	0.627
Retinol µg	−4.2 (6.2)	0.12	0.504	−10.1 (5.8)	0.99	0.082
Vitamin A µg	−2.7 (6.3)	0.05	0.667	−7.0 (6.3)	0.41	0.265
Vitamin C mg	−6.6 (5.1)	0.45	0.198	−4.9 (5.3)	0.28	0.352
Vitamin D µg	−0.1 (3.9)	0.00	0.968	−1.5 (4.2)	0.04	0.725
Vitamin E mg	−10.5 (7.5)	0.53	0.162	−16.2 (8.6)	1.15	0.061
Thiamin mg	−15.4 (5.5)	2.09	0.005 **	−12.5 (5.5)	1.61	0.026 *
Riboflavin mg	−27.4 (8.2)	2.96	0.001 **	−23.1 (8.8)	2.22	0.009 **
Niacin mg	−29.5 (7.9)	3.65	<0.001 ***	−10.1 (9.0)	0.41	0.261
Vitamin B6 mg	−17.0 (6.9)	1.63	0.014 *	−9.9 (7.6)	0.56	0.191
Vitamin B12 µg	−13.6 (6.3)	1.27	0.030 *	−6.9 (6.4)	0.38	0.279
Pantothenate mg	−24.2 (9.7)	1.68	0.013 *	−18.2 (9.7)	1.15	0.061
Biotin mg	−16.4 (7.7)	1.20	0.035 *	−20.5 (7.5)	2.39	0.007 *
Total Folate µg	−21.3 (8.1)	1.85	0.009 **	−13.3 (9.2)	0.68	0.148
Sodium mg	−8.2 (9.2)	0.22	0.369	−12.9 (10.2)	0.52	0.208
Potassium mg	−32.6 (9.8)	2.90	0.001 **	−26.0 (10.6)	1.91	0.015 *
Magnesium mg	−25.8 (9.7)	1.90	0.008 **	−23.2 (10.4)	1.59	0.027 *
Calcium mg	−15.2 (7.0)	1.28	0.030 *	−16.4 (7.6)	1.47	0.034 *
Phosphorus mg	−26.0 (9.7)	1.93	0.008 **	−28.6 (10.2)	2.51	0.005 **
Manganese µg	−19.9 (7.2)	2.04	0.006 **	−23.4 (8.1)	3.16	0.002 **
Iron mg	−16.3 (9.9)	0.73	0.100	−12.6 (10.3)	0.49	0.222
Zinc mg	−20.6 (9.4)	1.28	0.029 *	−11.8 (9.7)	0.48	0.226
Chlorine mg	−15.7 (8.9)	0.84	0.079	−17.8 (10.1)	1.01	0.078
Copper mg	−27.2 (9.0)	2.54	0.002 **	−19.3 (9.4)	1.37	0.040 *
Selenium µg	−0.6 (5.1)	0.00	0.902	−5.9 (5.8)	0.35	0.199

* *p* < 0.05, ** *p* < 0.01, *** *p* < 0.001. ^a^ Dietary includes beverages but excludes plain tap water. Total includes plain tap water as well. ^b^ SFA = saturated, MUFA = monounsaturated and PUFA = polyunsaturated fatty acids.

**Table 3:** Variables with significant relationships between log2 (nutrient density per MJ energy) and minutes of TIB in the second and third trimester, after adjusting for weight of dietary intake and welfare/disability status. Each nutrient density is considered separately.

Nutrient Density per MJ (All on log2 Scale)	Second Trimester (*n* = 370)		Third Trimester (*n* = 307)	
	Slope (SE)	*p*-Value	Slope (SE)	*p*-Value
Carbohydrate g/MJ	32.7 (16.3)	0.046	39.7 (22.8)	0.083
Sucrose g/MJ	16.6 (7.7)	0.033	5.9 (8.2)	0.472
SFA g/MJ	−1.1 (12.5)	0.928	−28.0 (13.5)	0.039
Thiamin mg/MJ	−11.2 (5.6)	0.048	−6.2 (5.6)	0.272
Riboflavin mg/MJ	−22.1 (9.8)	0.025	−5.2 (11.2)	0.645
Niacin mg/MJ	−25.1 (9.0)	0.010	6.6 (11.3)	0.556

**Table 4:** Multivariate regression for minutes of TIB in the second and third trimester combining confounders and nutrient variables.

	Second Trimester		Third Trimester	
	Slope (SE)	*p*-Value	Slope (SE)	*p*-Value
Welfare/Disability status	42.3 (13.1)	0.002	48.8 (13.8)	<0.001
Total hours of paid work	−0.69 (0.24)	0.005		
Number of preschool children	−21.5 (6.1)	0.001		
Weeks of gestation	−2.0 (0.9)	0.022		
Usually drank spirits before pregnancy	−26.0 (10.1)	0.011		
log2 (Dietary intake)	−31.2 (10.6)	0.003	−41.1 (11.3)	<0.001
log2 (SFA/MJ)			−28.0 (13.5)	0.039
log2 (Total sugars/MJ)	36.6 (14.5)	0.012		
log2 (Fructose/MJ)	−22.7 (9.0)	0.012		
log2 (Vitamin E/MJ)	28.1 (10.6)	0.008		
log2 (Potassium/MJ)	−46.8 (14.9)	0.002		
	R^2^ 17.1%		R^2^ 9.1%	

**Table 5:** Subjective frequency of difficulty sleeping in the second and third trimester.

	Second Trimester		Third Trimester	
Frequency	*n*	Percent	*n*	Percent
Never (coded 0)	178	35.3	81	18.1
Rarely (1)	85	16.9	41	9.2
Sometimes (2)	165	32.7	190	42.5
Often (3)	76	15.1	135	30.2

**Table 6:** Ordinal logistic regression for difficulty sleeping in the second trimester: (A) with nutrient intakes; (B) with food group frequencies.

Variable	Coefficient ^a^ (SE)	*p*-Value	95% CI for OR
(A) Model with nutrient intakes			
Week of gestation	−0.05 (0.02)	0.008	0.95 (0.92, 0.99)
Morning sickness severity Month 4 ^b^	−0.11 (0.04)	0.015	0.90 (0.82, 0.98)
Anxious	−0.21 (0.10)	0.033	0.81 (0.67, 0.98)
Depressed	−0.55 (0.12)	<0.001	0.58 (0.45, 0.73)
log2 (Niacin)	−0.43 (0.17)	0.011	0.65 (0.47, 0.91)
log2 (SFA)	−0.72 (0.26)	0.006	0.49 (0.29, 0.82)
log2 (MUFA)	0.80 (0.27)	0.004	2.21 (1.29, 3.81)
(B) Model with food group frequencies			
Week of gestation	−0.03 (0.02)	0.086	0.97 (0.94, 1.00)
Morning sickness severity Month 4 ^b^	−0.10 (0.04)	0.024	0.91 (0.83, 0.99)
Anxious	−0.21 (0.09)	0.023	0.81 (0.67, 0.87)
Depressed	−0.48 (0.12)	<0.001	0.62 (0.49, 0.78)
Daily Vegetable servings	0.24 (0.11)	0.029	1.27 (1.02, 1.57)
Daily Dairy servings	−0.12 (0.06)	0.042	0.89 (0.79, 1.00)

^a^ Negative coefficient implies increasing difficulty sleeping. ^b^ Morning sickness severity coded: None (0), Nausea occasionally (1), Nausea few hours/day (2), Nausea and vomit few hours/day (3), Nausea all day (4), Nausea all day, vomit occasionally (5), Vomit all day (6), Hospitalized (7).

**Table 7:** Ordinal logistic regression for difficulty sleeping in the third trimester: (A) with nutrient intakes; (B) with food group frequencies.

Variable	Coefficient ^a^ (SE)	*p*-Value	95% CI for OR
(A) Model with nutrient intakes			
Morning sickness continuing (1 = yes)	−0.52 (0.26)	0.049	0.60 (0.36, 1.00)
Morning sickness severity Month 4 ^b^	−0.18 (0.05)	<0.001	0.84 (0.76, 0.92)
Anxious	−0.21 (0.07)	0.002	0.81 (0.71, 0.93)
log2 (beta-carotene)	0.15 (0.08)	0.050	1.16 (1.00, 1.35)
log2 (vitamin B6)	−0.48 (0.15)	0.001	0.62 (0.46, 0.83)
log2 (weight of intake)	0.59 (0.23)	0.012	1.79 (1.13, 2.84)
(B) Model with food group frequencies			
Morning sickness continuing (1 = yes)	−0.60 (0.26)	0.020	0.55 (0.33, 0.91)
Morning sickness severity Month 4 ^b^	−0.16 (0.05)	0.001	0.85 (0.78, 0.94)
Anxious	−0.21 (0.07)	0.001	0.81 (0.71, 0.92)
Weekly Fruit code ^c^	0.36 (0.14)	0.008	1.43 (1.10, 1.87)
Weekly Dairy code ^c^	−0.36 (0.15)	0.020	0.70 (0.52, 0.94)

^a^ Negative coefficient implies increasing difficulty sleeping. ^b^ Morning sickness severity coded: None (0), Nausea occasionally (1), Nausea few hours/day (2), Nausea and vomit few hours/day (3), Nausea all day (4), Nausea all day, vomit occasionally (5), Vomit all day (6), Hospitalized (7). ^c^ Coded: None (0), Rarely (1), 1–2 times a week (2), 3–4 times a week (3), Daily (4).

## Data Availability

Due to confidentiality provisions, the data are not publicly available but can be examined by contacting the authors.
